# Shedding of Pandemic (H1N1) 2009 Virus among Health Care Personnel, Seattle, Washington, USA

**DOI:** 10.3201/eid1704.100866

**Published:** 2011-04

**Authors:** Meagan Kay, Danielle M. Zerr, Janet A. Englund, Betsy L. Cadwell, Jane Kuypers, Paul Swenson, Tao Sheng Kwan-Gett, Shaquita L. Bell, Jeffrey S. Duchin

**Affiliations:** Author affiliations: Centers for Disease Control and Prevention, Atlanta, Georgia, USA (M. Kay, B.L. Cadwell);; Public Health–Seattle and King County, Seattle, Washington, USA (M. Kay, P. Swenson, T.S. Kwan-Gett, J.S. Duchin);; Seattle Children’s Hospital, Seattle (D.M. Zerr, J.A. Englund, S.L. Bell);; University of Washington, Seattle (J. Kuypers, T.S. Kwan-Gett, J.S. Duchin)

**Keywords:** Influenza, virus shedding, pandemic (H1N1) 2009, viruses, PCR, health care personnel, outbreak, Washington, research

## Abstract

The Centers for Disease Control and Prevention (CDC) recommends that health care personnel (HCP) infected with pandemic influenza (H1N1) 2009 virus not work until 24 hours after fever subsides without the use of antipyretics. During an influenza outbreak, we examined the association between viral shedding and fever among infected HCP. Participants recorded temperatures daily and provided nasal wash specimens for 2 weeks after symptom onset. Specimens were tested by using PCR and culture. When they met CDC criteria for returning to work, 12 of 16 HCP (75%) (95% confidence interval 48%–93%) had virus detected by PCR, and 9 (56%) (95% confidence interval 30%–80%) had virus detected by culture. Fever was not associated with shedding duration (p = 0.65). HCP might shed virus even when meeting CDC exclusion guidelines. Further research is needed to clarify the association between viral shedding, symptoms, and infectiousness.

Health care personnel (HCP) with influenza infections can transmit virus to patients. This finding is of particular concern for patients with underlying medical conditions and places them at risk for serious influenza infections. Understanding the duration of shedding of pandemic (H1N1) 2009 virus detected by rapid culture and real-time reverse transcription–PCR (RT-PCR) among HCP is useful in developing infection prevention measures for the health care setting.

The Centers for Disease Control and Prevention (CDC) created guidelines for infection control in health care settings to prevent influenza transmission from infected HCP to patients and other HCP (*1*). These guidelines for the 2009 influenza season (2009 CDC criteria) recommend that HCP who have a fever and respiratory symptoms stay home from work for 24 hours after fever subsides without the use of fever-reducing medications. HCP who do not have a fever are permitted to work if they use appropriate infection control practices (*1*). CDC also recommends that HCP who are caring for severely immunocompromised patients (e.g., patients with hematopoietic stem cell transplantations) be considered for temporary reassignment or be excluded from work for 7 days from symptom onset or until resolution of symptoms, whichever period is longer (*1*). Earlier return to work is permitted for HCP who are caring for patients with lesser degrees of immune system compromise who also might be at increased risk for complicated influenza infections.

A limited number of studies have described the duration of pandemic (H1N1) 2009 virus shedding among healthy persons, as estimated by the presence of viral RNA detected by real-time RT-PCR or viable virus detected by culture. A study by the US Air Force demonstrated that viable virus was present in 24% of nasal wash samples from infected military trainees 7 days after symptom onset (*2*). To et al. reported that virus was undetectable by culture 5 days after symptom onset or by real-time RT-PCR at 8 days among 21 of 22 hospitalized patients treated with oseltamivir (*3*). Among household cases, Suess et al. reported mean shedding durations by real-time RT-PCR for treated and untreated patients of 5.7 days and 7.1 days, respectively (*4*).

On September 27, 2009, Public Health–Seattle and King County (PHSKC) in Seattle, Washington, was notified of an outbreak of pandemic (H1N1) 2009 among young, otherwise healthy HCP (medical residents) who had attended a work retreat at hospital A during September 21–25. We conducted an investigation to characterize the influenza outbreak and describe viral load changes, shedding duration, and the association between these factors and fever.

## Methods

On September 27, 2009, after the hospital infection prevention office at PHSKC was notified about a retreat participant with acute influenza A, all HCP who had attended the work retreat were contacted. HCP who were scheduled to be working were assessed to determine the presence of respiratory symptoms. All HCP with any respiratory symptoms were tested for influenza infection by direct fluorescent antibody (DFA) testing or real-time RT-PCR at Hospital A; negative DFA results were confirmed by real-time RT-PCR (*5*). All viruses were subtyped.

### Descriptive Data

All retreat attendees were informed of the study and asked to participate. Participation in the study was voluntary. Attendees who agreed to participate completed an online questionnaire that included date of birth, sex, race/ethnicity, job title, underlying health conditions, time spent at the retreat, treatment or prophylaxis compliance, and symptoms. The questionnaire was distributed through SurveyGizmo (Widgix, LLC, Boulder, CO, USA), an online vendor with a password-protected website that had an existing Health Insurance Portability and Accountability Act of 1996 business associate agreement with PHSKC. Demographic information was obtained from employee health records for nonrespondents.

HCP met the case definition if they had attended the retreat for any period during September 21–25, 2009, and had laboratory evidence of influenza infection by DFA or real-time RT-PCR from a specimen obtained during September 26–28. All persons meeting the case definition were asked to record symptoms and oral temperatures daily for 2 weeks or until no longer symptomatic. Measured fever was characterized as an oral temperature >100.5°F during illness. Those persons who reported a fever but did not measure their temperature or measured an oral temperature <100.5°F were classified as having subjective fever.

### Specimen Collection

To examine viral shedding quantitatively, we began sampling of nasal washes on September 30. Self-administered nasal washes were performed every Monday, Wednesday, and Friday until 2 consecutive negative real-time RT-PCR results were documented. Instructions on how to perform a nasal wash were provided in writing and demonstrated to each participant individually. Participants were instructed to quickly instill 5 mL of normal saline without preservatives into 1 nostril, immediately tip their head down, and blow their nose forcefully into a paper cup. Using a 3-mL syringe, participants transferred half of the sample to a plastic specimen vial and half to a container of viral transport media. If <2 mL was obtained, the process was repeated by using the other nostril. Samples were transported on ice to our laboratory on the day they were obtained. Participants not available for sampling on the first day of collection were entered into the study on the subsequent sampling day.

### Laboratory Testing

Each nasal wash specimen was tested by rapid culture and real-time RT-PCR. Rapid culture was performed by the PHSKC Laboratory to detect viable virus. In this method, 0.2 mL of specimen was injected into 1 shell vial of R-Mix cells (Diagnostic Hybrids, Athens, OH, USA). The vial was centrifuged for 1 hour at 700 × *g* and incubated for 20–24 hours at 36°C. After incubation, the coverslip was stained with influenza A monoclonal antibody by using immunofluorescence (*6*). Viral load was quantified by real-time RT-PCR at the University of Washington Virology Laboratory as the influenza virus RNA concentration per milliliter of sample (*5*). Nasal wash samples were not quantified by culture.

### Statistical Analysis

Questionnaire responses, employee health record data, symptom log, and laboratory data were entered into a spreadsheet and analyzed by using SAS version 9.1 (SAS Institute, Inc., Cary, NC, USA). Frequencies were calculated for categorical variables, and Fisher exact tests were used to examine associations between categorical variables. Medians were calculated for continuous variables. An accelerated failure time model with a Weibull survival function was used to assess the effect of test type (i.e., real-time RT-PCR or rapid culture), presence of measured or subjective fever, and viral load (of the first available serial sample) on duration of viral shedding. This model accounts for interval censoring present in data and uses a range for shedding duration. This range is based on the fact that initiation of shedding was considered to be the day of symptom onset, and cessation of shedding was considered to range from the collection date of the last positive test result to collection date of the first of 2 consecutive negative test results. Shedding duration was calculated as the number of days between initiation and cessation of shedding (*7*). For participants who showed negative results on the first day of nasal wash sampling, cessation was considered to range from symptom onset to obtaining the first of 2 consecutive negative test results. To evaluate whether fever was a predictor of viral load, a linear regression model was fit by using viral load as the dependent variable. A p value <0.05 was considered statistically significant in all analyses.

### Human Subjects Review

This study was conducted as part of an outbreak investigation by PHSKC in collaboration with Hospital A. The study was classified as nonresearch by the Washington State Institutional Review Board and a CDC human subjects review coordinator.

## Results

The retreat occurred from 8:00 am to 5:00 pm daily for 5 days, with optional evening social activities. Forty-six persons participated in retreat activities. Fourteen persons were facilitators for the retreat, did not attend all of the retreat events, and typically participated for <4 hours/day when attending. The remaining 32 core participants attended all 5 days of the retreat and participated in evening activities. Most core participants also stayed overnight in 1 cabin on Thursday, September 24.

On Friday, September 25, the last day of the retreat, the first retreat participant with documented illness experienced a cough. The following day, this person had fever and was DFA positive for influenza A. By Monday, September 28, active surveillance of the 46 retreat attendees by Hospital A staff identified 20 persons with respiratory symptoms; 19 were core participants and 1 was a facilitator. Influenza A virus was detected by DFA or real-time RT-PCR in 17 symptomatic retreat attendees; all were core participants. All viruses from these 17 persons were confirmed by real-time RT-PCR to be pandemic (H1N1) 2009 virus. Thus, of the 32 core participants at the retreat, 17 (53%) were virus positive; 7 of the 17 had measured fever and cough or sore throat. The other 3 participants with respiratory symptoms were virus negative; all were afebrile.

All HCP who were virus positive were treated with oseltamivir, 75 mg orally 2×/d for 5 days, within 48 hours of symptom onset. In accordance with CDC recommendations at that time, ill HCP were excluded from work for 7 days after symptom onset. Core participants and facilitators who did not become symptomatic or positive for influenza A were administered antiviral chemoprophylaxis (oseltamivir, 75 mg orally, 1×/d for 10 days) and were not excluded from work.

Questionnaires were completed by 45 (98%) of 46 retreat attendees, including 16 of 17 persons who were positive for pandemic (H1N1) 2009 virus infection. Sixteen infected HCP provided serial nasal wash samples and completed a symptom log for the duration of their illness. One virus-infected participant did not complete a questionnaire but did provide serial nasal wash specimens. Another participant who was virus positive completed a questionnaire but did not provide serial nasal wash specimens.

HCP infected with pandemic (H1N1) 2009 virus ranged in age from 26 to 33 years (median 28.5 years). Sixteen (94%) infected HCP were women compared with 67% of core attendees who did not meet the case definition (p = 0.06). Three (18%) infected HCP had an underlying medical condition compared with 1 (7%) of 15 core attendees who did not meet the case definition (p = 0.60). Of those infected HCP who completed the questionnaire, cough, myalgias, and headache were the most commonly reported symptoms (Table), with >2 of these symptoms reported by all 16 HCP, and all 3 symptoms reported by 13 (81%) HCP. According to questionnaire data or employee health records of all 17 infected HCP, fever >100.5°F was measured by 7 (41%) infected HCP, and an additional 5 (29%) infected HCP reported subjective fever. HCP who reported fever typically documented the fever only during the first 1–2 days of illness, and no one reported measuring an oral temperature >100.5°F for >4 days. All HCP reported completing antiviral treatment as prescribed, and no deaths or hospitalizations occurred.

**Table:** Signs and symptoms among 16 health care personnel infected with pandemic (H1N1) 2009, Seattle, Washington, USA*

Sign or symptom	No. (%) persons
Cough	16 (100)
Myalgia	15 (94)
Headache	15 (94)
Chills	12 (75)
Sore throat	10 (63)
Measured fever†	7 (44)
Subjective fever‡	5 (31)
Diarrhea	3 (19)
Vomiting	3 (19)

*Information on all symptoms was not available for 1 infected person. †Oral temperature >100.5°F during illness. ‡Fever but no measurement of an oral temperature >100.5°F during illness.

Among 7 HCP who measured a fever >100.5°F during their illness, 5 (71%) were virus positive by real-time RT-PCR and 3 (43%) were positive by rapid culture >24 hours after defervescence, when they met 2009 CDC criteria for returning to work. Of the 9 HCP who did not measure a fever >100.5°F during their illness and therefore did not meet CDC exclusion criteria, 7 (78%) were positive by real-time RT-PCR; 6 (67%) were also positive by rapid culture at the time of collection of the initial serial nasal wash sample >3 days after symptom onset. Samples from 12 (75%) of 16 HCP (95% confidence interval 48%–93%) were positive by real-time RT-PCR, and samples from 9 (56%) of 16 HCP (95% confidence interval 30%–80%) were positive by rapid culture at the time they met 2009 CDC criteria for returning to work (24 hours after defervescence). From the onset of symptoms, the duration of viral shedding determined by real-time RT-PCR results ranged from 3 to 13 days compared with 3–10 days by rapid culture results. Among 7 infected HCP, virus was detected by real-time RT-PCR and rapid culture for the same number of days. Among 8 HCP, virus was detected ≈2 days longer by real-time RT-PCR than by rapid culture. In 1 HCP, virus was detected by rapid culture 3 days longer than by real-time RT-PCR.

**Figure 1:**
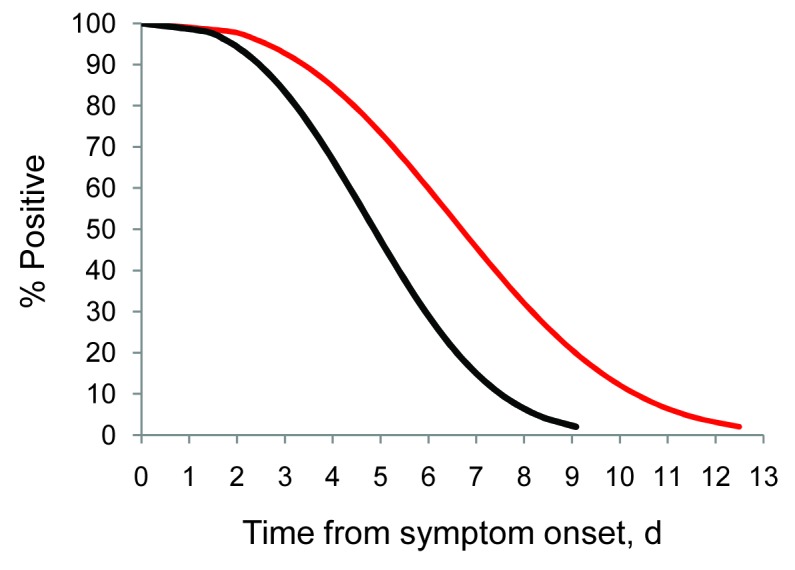
Survival analysis model of pandemic (H1N1) 2009 virus shedding over time among infected health care personnel, Seattle, Washington, USA. Survival curves were modeled on data for 16 persons who became infected with pandemic (H1N1) 2009 virus after attending a work retreat in September 2009. A negative test result by rapid culture (black line) or real-time reverse transcription–PCR (red line) was the event of interest. Shedding duration determined by using real-time reverse transcription–PCR was significantly longer than that determined by rapid culture (p = 0.02).

In our initial model using data from all 16 participants, which included test type (real-time RT-PCR vs. rapid culture), presence of fever (measured and subjective), and viral load as predictors of shedding duration, test type was the only significant variable (p = 0.02). Our results indicated no association between fever (measured and subjective) and shedding duration (p = 0.65) or between viral load and shedding duration (p = 0.74). Our final model, which included only test type as a predictor of shedding duration, indicated that shedding duration was significantly different between rapid culture and real-time RT-PCR (Figure 1). Shedding duration measured by rapid culture was 0.73× higher than shedding duration measured by real-time RT-PCR (p = 0.02). Eight of 16 HCP remained positive by rapid culture 4.8 days from symptom onset compared with 6.6 days by real-time RT-PCR.

**Figure 2:**
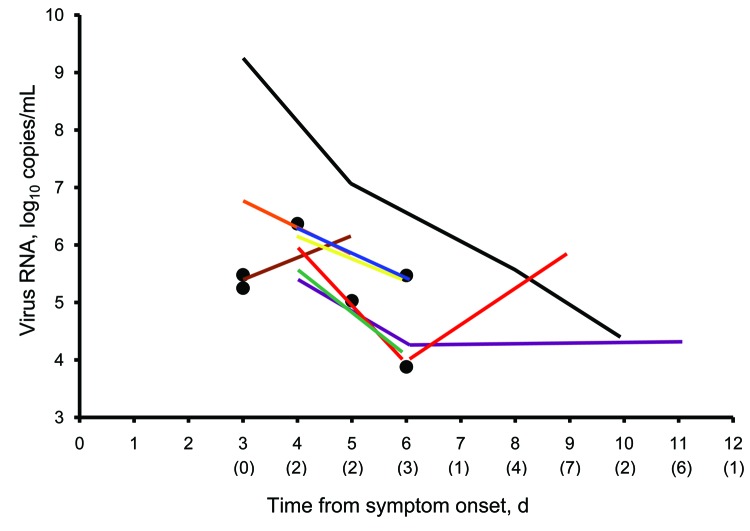
Virus RNA concentrations over time among health care personnel infected with pandemic (H1N1) 2009 virus, Seattle, Washington, USA. Each colored line represents a virus RNA concentration for an infected person tested from symptom onset until the first of 2 consecutive negative results by real-time reverse transcription–PCR (RT-PCR) for pandemic (H1N1) 2009 virus. Persons who had virus detected by real-time RT-PCR only once are indicated by solid circles. The lower detection limit of the real-time RT-PCR was 3 log_10_ copies/mL. Numbers of persons with virus RNA concentrations below the detection limit for each day after symptom onset are shown in parentheses below the x-axis. Sixteen infected persons were receiving oseltamivir.

An overall decrease in viral RNA concentration with time was measured and quantified by real-time RT-PCR (Figure 2). The person with the highest concentration of viral RNA on the first day of serial sampling also had one of the longest shedding durations by real-time RT-PCR. However, viral load was not a significant predictor of shedding duration in our analysis. Measured fever and subjective fever were not correlated with viral load (p = 0.41 and 0.12, respectively).

## Discussion

We describe a pandemic (H1N1) 2009 outbreak with a high attack rate that involved relatively young, healthy HCP after a hospital-associated retreat. Because of the single source of infection in an enclosed environment, we were able to prospectively monitor study participants to document duration of clinical symptoms and laboratory parameters. Infected HCP often did not report a fever during their illness. Virus infection was documented by real-time RT-PCR and rapid culture for prolonged periods, even though all infected HCP were treated with oseltamivir within 24–48 hours of illness onset. The shedding duration measured by real-time RT-PCR was significantly longer than that measured by rapid culture.

A total of 17 (53%) of 32 participants who attended all 5 days of the retreat became infected with virus. This rate of secondary transmission among core attendees is higher than that of other reports of secondary attack rates, which have been <35% (*2**,**8**–**10*). The high attack rate we observed might be explained by a susceptible population without immunity to pandemic (H1N1) 2009 virus, active surveillance for infection, or by prolonged close contact among participants, including sharing a cabin. Although viral load of the index case on day 1 of illness was not quantified, the initial viral load (5.03 log_10_ RNA copies/mL) in this person on day 5 of illness was higher than the day 5 mean and median viral loads reported in other studies (*4**,**11**,**12*), which suggested that a high viral load might have contributed to the high attack rate in this outbreak. These findings highlight the potential for rapid spread of influenza among populations with ongoing close contact. In the absence of an available vaccine, handwashing and respiratory hygiene practices (e.g., cough etiquette and exclusion or isolation) should be emphasized in settings where persons might be in prolonged close contact with one another (e.g., hospitals, schools, or shelters).

This study had several limitations. Although all persons received standardized instructions to minimize variation in collection methods, nasal washes were self-collected and had the potential for variability in collection technique. Self-collection of nasal washes might have led to underestimation of presence or quantity of influenza virus. Other studies have demonstrated increased sensitivity with nasal washes, compared with nasal swabs, in respiratory virus detection (*4**,**13*), and we believe that self-collection of nasal washes likely increased compliance with study procedures.

For logistical reasons, persons were not tested daily, which affected our ability to more precisely define duration of shedding. We chose to use an accelerated failure time model with a Weibull survival function in our analysis because this method accounts for interval censoring. However, our model did not account for the paired nature of the data, in that each sample was tested by rapid culture and real-time RT-PCR. Our unpaired analysis likely led to an overly conservative p value, indicating that a statistically significant p value in our analysis also might have been detected by an analytic technique that accounts for pairing. The small sample size of this study might have decreased the power to detect associations observed in previous studies even if they existed, such as an association between fever and shedding duration or fever and viral load (*12*).

In addition, viral load was not measured on the day of symptom onset in our study but began on the first day that the participant provided serial nasal wash specimens. This delayed measurement might have affected the ability to detect associations between viral load and shedding duration and between fever and viral load. Furthermore, our findings should be considered in light of the fact that all participants were treated with antiviral medication, which might have affected relationships between symptoms, shedding duration, and viral load. Previous research has shown that oseltamivir reduces the duration of symptoms and influenza virus shedding (*14**,**15*). We did not prospectively ask about the use of fever-reducing medications among infected HCP, and therefore we were unable to definitively assess the potential effect of their use on our findings. Considering that in 6 of 7 HCP who had measured fever during their illness, the fever had resolved at least 36 hours before testing was performed, we do not believe that prospective assessment of the use of fever-reducing medications would have changed our conclusions.

Most characteristics of infection were similar to those of previous reports of pandemic (H1N1) 2009 virus infections (*16**–**18*), including an outbreak among HCP in Kenya (*8*). Approximately one third of infected HCP in our study did not have measured or subjective fever during their infection. This observation is consistent with those of previous reports (*19**,**20*). Those HCP who had an oral temperature >100.5°F typically documented fever only during the first 1–2 days of infection, although this finding might be explained by oseltamivir treatment that resulted in a shorter duration of symptoms. Fever has been demonstrated in some studies (*12**,**21*) to correlate with influenza virus shedding. However, we observed that fever was not statistically associated with viral shedding duration (i.e., those HCP who had a fever were not more likely to be positive by real-time RT-PCR or rapid culture longer than those HCP who never had a fever). The association between fever and virus shedding duration might have been modified by antiviral treatment; the small sample size also might have affected the power to detect an association between fever and shedding duration if one existed. In our study, 50% of participants remained positive at 6.6 days by real-time RT-PCR, which is consistent with the average shedding duration reported by Suess et al. (6.6 days) (*4*), but considerably longer than 4.5 days reported by Carrat et al. (*11*).

Most HCP in our study were shedding virus that was detected by real-time RT-PCR and rapid culture after they met CDC criteria for returning to work. Our findings support those of a study that found that persons with influenza infections who shed virus cannot be reliably identified by using fever alone (*11*). These results raise essential considerations regarding exclusion policies for infected HCP. Because febrile and afebrile HCP had similar virologic shedding durations and viral loads, the absence of influenza by real-time RT-PCR or culture might be preferable to the absence of fever as a criterion for HCP who are returning to work in settings where they place others at high risk. A critical caveat is that positive influenza real-time RT-PCR or rapid culture results do not necessarily indicate that the patient is capable of transmitting virus to others. Although reported transmission dynamics of pandemic (H1N1) 2009 virus are similar to those of seasonal influenza viruses (*4*), the generalizability of our findings to seasonal influenza viruses is uncertain.

Our findings, although limited to pandemic (H1N1) 2009 virus infections, suggest that HCP who return to work after other influenza infections might continue to excrete viable virus and reinforce the need for adherence to infection control measures to prevent transmission in the workplace. All HCP who return to work after influenza infection should practice frequent hand and respiratory hygiene and cough etiquette. Because many HCP were afebrile and others might still be infectious >24 hours after defervescence, our results support the overriding need for influenza vaccination of HCP as the preferred prevention method in health care settings. Further research is needed to determine whether detection of influenza virus and clinical symptoms correlates with infectivity with or without antiviral use.
